# Plasma Proteomes Can Be Reidentifiable and Potentially Contain Personally Sensitive and Incidental Findings

**DOI:** 10.1074/mcp.RA120.002359

**Published:** 2021-01-11

**Authors:** Philipp E. Geyer, Sebastian Porsdam Mann, Peter V. Treit, Matthias Mann

**Affiliations:** 1 Department of Proteomics and Signal Transduction, Max Planck Institute of Biochemistry, Martinsried, Germany; 2 Faculty of Health Sciences, NNF Center for Protein Research, University of Copenhagen, Copenhagen, Denmark; 3 OmicEra Diagnostics GmbH, Planegg, Germany; 4 Department of Media, Cognition and Communication, University of Copenhagen, Copenhagen, Denmark; 5 Uehiro Center for Practical Ethics, Oxford University, Oxford, UK

**Keywords:** ethics, biomarker discovery, clinical proteomics, alleles, CRP, C-reactive protein, CSF, cerebrospinal fluid, FDA, Food and Drug Administration, FDR, false discovery rate, GDPR, General Data Protection Regulation, hCG, human chorionic gonadotropin, HIPAA, Health Insurance Portability and Accountability Act, QTL, quantitative trait loci, MS, mass spectrometry, PSG, pregnancy-specific glycoprotein, PZP, pregnancy zone protein, SAA1, serum amyloid alpha 1, SNP, single nucleotide polymorphism

## Abstract

The goal of clinical proteomics is to identify, quantify, and characterize proteins in body fluids or tissue to assist diagnosis, prognosis, and treatment of patients. In this way, it is similar to more mature omics technologies, such as genomics, that are increasingly applied in biomedicine. We argue that, similar to those fields, proteomics also faces ethical issues related to the kinds of information that is inherently obtained through sample measurement, although their acquisition was not the primary purpose. Specifically, we demonstrate the potential to identify individuals both by their characteristic, individual-specific protein levels and by variant peptides reporting on coding single nucleotide polymorphisms. Furthermore, it is in the nature of blood plasma proteomics profiling that it broadly reports on the health status of an individual—beyond the disease under investigation. Finally, we show that private and potentially sensitive information, such as ethnicity and pregnancy status, can increasingly be derived from proteomics data. Although this is potentially valuable not only to the individual, but also for biomedical research, it raises ethical questions similar to the incidental findings obtained through other omics technologies. We here introduce the necessity of—and argue for the desirability for—ethical and human-rights-related issues to be discussed within the proteomics community. Those thoughts are more fully developed in our accompanying manuscript. Appreciation and discussion of ethical aspects of proteomic research will allow for deeper, better-informed, more diverse, and, most importantly, wiser guidelines for clinical proteomics.

## Highlights

• Plasma proteomes can be reidentified based on protein expression levels.
• Plasma proteomes can be reidentified based on variant peptides.
• Plasma proteomes can contain actionable and nonactionable incidental findings.
• Plasma proteomes can contain sensitive information such as pregnancy status.

## Graphical Abstract

**Figure:**
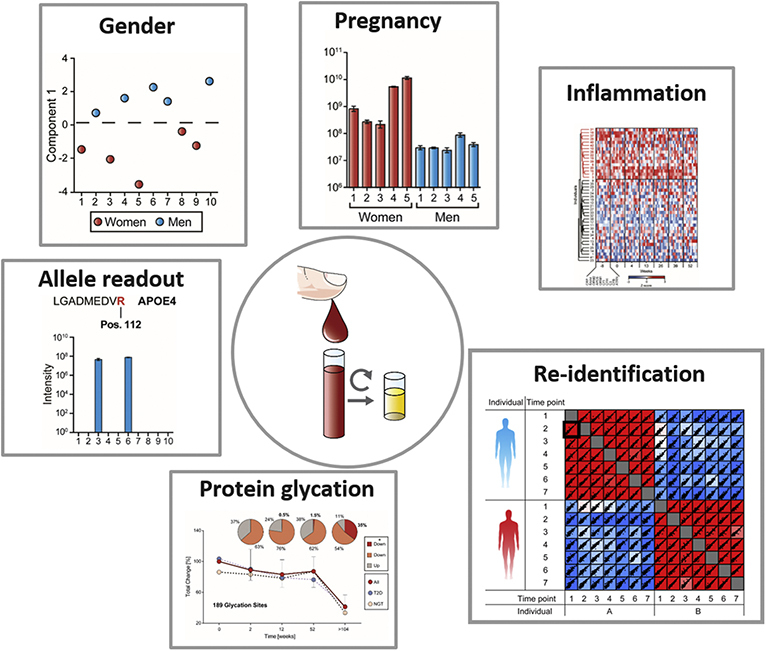

## In Brief

Due to its unbiased nature, proteomics may raise ethical and regulatory concerns. Plasma proteome samples can be reidentified given genomic information. Furthermore, plasma proteomes contain information of potential sensitive nature. Incidental findings can help diagnose unrelated but actionable disease states.

Omics technologies can characterize biological materials, leading to a wealth of information useful for addressing a broad range of scientific and medical questions. Genomics has benefited from rapid technological progress over many decades, and large-scale DNA analysis now increases our knowledge of genetic diversity and the relation of genes to various phenotypical and disease-relevant traits (1). Genomic data are usually acquired in a broad and untargeted manner. Typically, many genes are assayed simultaneously. Some of these could contain individually identifiable and sensitive information, raising several ethical questions. The relatively long-lasting and widespread application of genomics in research and medicine has led to numerous and sometimes acrimonious debates concerning what to do with such information, which in turn has resulted in guidelines and frameworks (2, 3, 4). However, analogous discussions have not yet been fielded in proteomics or metabolomics. Partly this is because the ability to analyze a large number of human samples at great proteomic depth is a comparatively new and resource-intensive development, which as of this writing still requires highly specialized technology.

In contemporary medical practice, the majority of diagnostic decisions are based on tests quantifying biological parameters, generally referred to as “biomarkers” (5). The foremost bodily fluids used for this purpose are the blood (whole, plasma, or serum), urine, and cerebrospinal fluid (CSF). Of the various classes of clinically measured parameters such as cells, electrolytes, DNA, RNA, and small molecules, most tests target proteins (6). Thousands of these proteins circulate throughout the body and their levels report on a wide variety of systemic diseases, organ damage, and general health status (7, 8, 9).

From an analytical biochemistry perspective, the biomarker discovery task has traditionally consisted of accurately measuring the level of one or a few proteins in disease and control cohorts and then developing a robust clinical test—typically involving an antibody against a specific protein. Considering the fundamental role of laboratory tests, it is remarkable that most biomarkers were discovered more than 20 years ago and often lack specificity or sensitivity and that only a very small number of the more than 14,000 human diseases have corresponding tests (10).

Proteins control and execute the vast majority of biological processes, and mass spectrometry (MS)-based proteomics is the technology of choice to investigate the entirety of all proteins in a biological system—its proteome (11, 12). MS-based proteomics has continuously developed over the past 20 years, enabling the holistic, detailed, and quantitative investigation of diverse biological systems. Discovery of new and potentially better biomarkers and biomarker panels is facilitated by these rapid developments in MS-based proteomics, promising widespread medical applications (6, 13).

Traditionally, proteomics biomarker studies have analyzed small sample numbers in depth to discover one or a few potential biomarkers that were then to be validated in larger cohorts. In contrast, we have developed a “rectangular approach for biomarker discovery,” in which all samples from large-scale cohorts are analyzed in as much depth as possible in discovery and validation cohorts together (6). The goal is to derive panels of regulated proteins that contain much more information than is reflected in the level of any single protein. Over the last few years, we have focused on technological developments enabling the analysis of large-scale plasma proteomic cohorts with a robust and automated pipeline (14) and have already analyzed clinical studies with over a thousand samples (15, 16, 17).

It will soon be possible to collect large-scale and increasingly comprehensive proteomics data sets, and this ability will not long be restricted to only a few specialized laboratories. Clinical proteomics is already benefitting from rapid advances in information technologies, including machine learning and big data analytics. The information that can be extracted from such powerful data sets has myriad applications. Clearly, the expanding data volume, scope, and quality of clinical studies analyzed by MS-based proteomics raise ethical issues, as they have in other fields. For instance, a plasma proteomic measurement may be used to uniquely identify a person if matching genomic information is available. In addition, such a measurement may contain incidental findings—findings unrelated to the primary aim of the study or procedure, but which may still contain information relevant to health or well-being. This potential is well recognized in other diagnostic fields. In a meta-analysis of tens of thousands of asymptomatic persons receiving body or brain MRI revealed a potentially serious incidental findings rate of 3.9% and another found a median clinically significant rate of 17% (18, 19). It has been pointed out that incidental findings, “which can occur in large numbers from genomic sequencing, are a potential barrier to the utility of this new technology due to their relatively high prevalence and the lack of evidence or guidelines available to guide their clinical interpretation” (20).

We argue that clinical proteomics, too, will soon face these challenges. Researchers and clinicians will have to deal with personally sensitive and incidental findings. How they should go about this task is a discussion that must be had. It is also a discussion that involves applied ethics and bioethical principles—topics that the proteomics community is currently not prepared for. As a first step in this direction and to enable our community to begin an open discussion about proteome ethics, we describe some ethical implications of proteomics data. Below, we reanalyze a previous plasma proteomics study (15) to investigate whether and to what extent ethically relevant information can be extracted. We show that samples can uniquely be assigned to individuals by both the individual-specific levels of plasma proteins and their individual-specific allele variations (coding SNPs). Furthermore, plasma proteomes inherently report on a broad range of health and disease parameters such as cardiovascular and metabolic risks. We discuss what would have to be stripped from results obtained in order only to retain narrowly disease-relevant information. While this might be necessary in certain diagnostic settings, it also negates one of the principal attractions of plasma proteomics. In an accompanying paper (21), we introduce bioethical and human rights principles that instead argue for deriving the maximum information and therefore benefit from clinical proteomics data for research, disease diagnosis, and general health and well-being.

## Experimental Procedures

### Identification of Individual-Specific Alleles

All peptides from the variant FASTA file analysis were filtered to generate a set of reliable peptides suitable to separate individuals. For this purpose, we filtered the data set for peptides that were present at least once in six out of the seven time points in at least one individual. We excluded all peptides that were always or never identified as they do not contain information that could be used to distinguish between individuals. Next, we filtered for peptides that had at least one overlapping peptide from another allele. This resulted in 83 peptides. Peptides containing a missed cleavage site of Arginine or Lysine will contain the same information as their fully cleaved form. Hence, the information of the presence of the allele was only counted once, resulting in a set of 67 peptides. This set also contains alleles that were very randomly distributed with very high variation. The peptides with the highest variation were excluded. If the number of the identified peptides in the study was ten times larger than the sum of the peptides that were identified six or seven times within an individual, both alleles of the same gene were excluded, resulting in 53 peptides for the analysis.

### Data Analysis

MS raw files were analyzed by MaxQuant software, version 1.6.1.9 (22), and peptide lists were searched against the human Uniprot FASTA databases. A regular FASTA file was downloaded from the UniProt database in May 2019 (https://www.uniprot.org/). Variant sequence entries were downloaded in text format from the UniProt database in May 2019 (ftp://ftp.uniprot.org/pub/databases/uniprot/current_release/knowledgebase/taxonomic_divisions/). The Swissknife PERL module (http://swissknife.sourceforge.net/docs/) with the varsplic PERL script from ftp://ftp.ebi.ac.uk/pub/software/uniprot/varsplic/varsplic.pl was applied to generate the variant text formats for single sequences. The output produced includes the sequence for the variants.

A contaminant database generated by the Andromeda search engine (23) was configured with cysteine carbamidomethylation as a fixed modification and N-terminal acetylation and methionine oxidation as variable modifications. We set the false discovery rate (FDR) to 0.01 for protein and peptide levels with a minimum length of seven amino acids for peptides, and the FDR was determined by searching a reverse database. Enzyme specificity was set as C terminal to arginine and lysine as expected using trypsin and LysC as proteases. A maximum of two missed cleavages were allowed. Peptide identification was performed with an initial precursor mass deviation up to 7 ppm and a fragment mass deviation of 20 ppm. All proteins and peptides matching the reversed database were filtered out.

All bioinformatics analyses were performed with the Perseus software of the MaxQuant computational platform (22, 24).

## Results

### Individuals Can Be Identified by Protein Levels in Blood Plasma

In clinical studies, samples are usually blinded and pseudonymized in order to avoid bias by the experimenter and to protect study participants from potentially sensitive findings. In this way, results from a sample cannot directly be tied to a person, *i.e.*, unusually high cholesterol levels could not be used to deny insurance. In our previous work, we noticed that the levels of hundreds of plasma proteins varied much more between participants than within the same participant over time (15). We therefore speculated that the “individual-specific” levels of many plasma proteins together could enable association of a given plasma proteome sample to a previous measurement on the same person. Although this would prevent the pervasive problem of sample mix-up (25), it could conceivably raise ethical issues with regard to reidentification of participants.

**Fig. 1:**
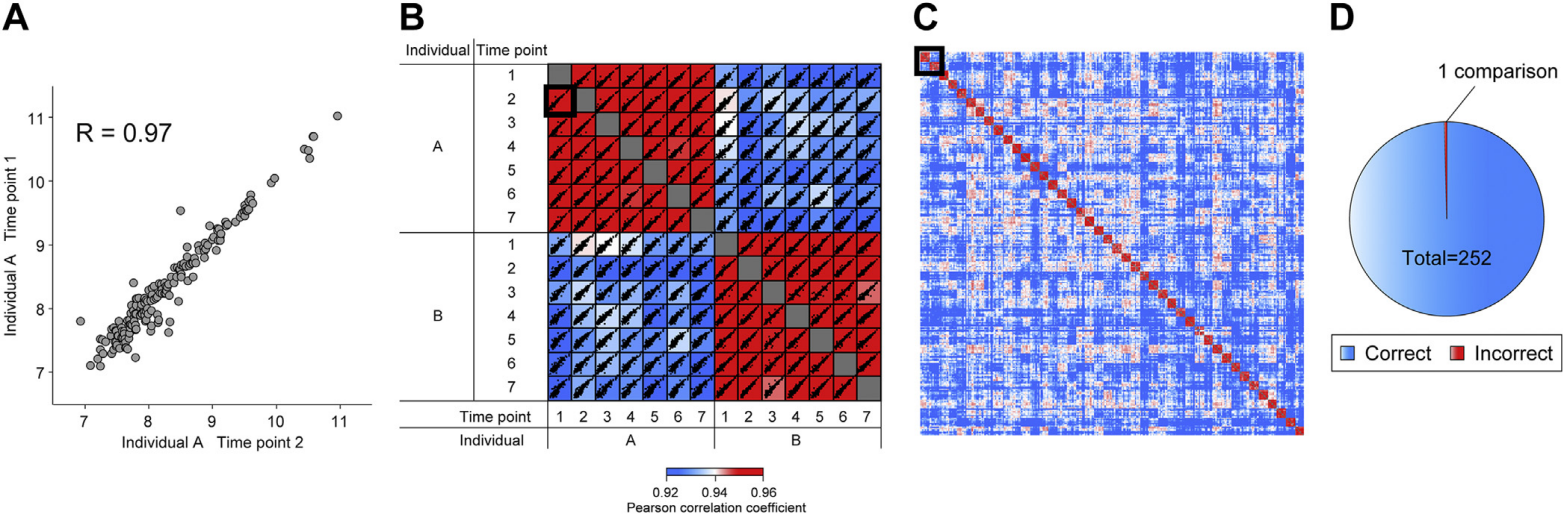
**Identifying participants in a longitudinal study by correlation of individual-specific proteins.***A*, correlation of individual-specific proteins of time points 1 and 2 of individual *A*. *B*, seven longitudinal samples of two individuals, *A* and *B*, are correlated with each other (Pearson correlation coefficient is color-coded, with color bar below). The comparison displayed in (*A*) is highlighted by a *black frame*. *C*, cross-correlated individual-specific proteins of all samples of the weight loss study. The correlation matrix shown in (*B*) is highlighted by a *black frame* (Pearson value coded according to the same color bar). *D*, identification of individuals by correlating individual-specific proteins. Proteomes of the reference time point were compared with all other time points in turn. The percentage of correctly and incorrectly assigned participants is color-coded.

To test our hypothesis, we reinvestigated a plasma proteomics weight loss study in which samples of 42 individuals were obtained over 1 year (15). We defined a protein as “individual-specific” if its level in a participant was more than 1.5-fold different from the population median for at least a quarter of all study participants, and it had a coefficient of variation below 20% over time. In our study, 71% of all proteins fulfilled these criteria. When we compared the levels of individual-specific proteins over time, we found that these intraindividual correlations were much higher than the correlations of the same or different time points between two different persons (91 different correlation values; median Pearson R = 0.971 within and 0.926 between the two individuals in the example) (Fig. 1, *A* and *B*).

Global correlation of the 294 weight loss samples resulted in a matrix of 43,071 values (Fig. 1*C*). Pearson correlation coefficients of these individual-specific proteins had much higher intraindividual correlations even over a whole year, compared with interindividual correlations (Fig. 1*B*). Across the entire study, the median intraindividual correlation was 0.974 and the interindividual correlation was 0.928.

We next tried to identify individuals solely by the Pearson correlation coefficients of their individual-specific proteins. For this purpose, we defined the preweight loss time point as a reference and asked whether the others could be uniquely related to it *via* their plasma proteome correlations. Out of these 252 comparisons, all but one were assigned to the correct individual (Error rate of 0.4%; Fig. 1*D*). Even in the one misassignment (possibly caused by experimental issues; Experimental Procedures), the correct individual was ranked second. Moreover, highly individual-specific proteins such as the apolipoprotein(a) can be more than 100-fold different between individuals, but very constant over time. In the case of apolipoprotein(a), this can be explained by the genetically determined number of so-called Kringle domains affecting the concentration of this protein. Such proteins have a higher value for identifying or excluding an individual. In general, quantitative trait loci (pQTLs) link protein levels and genetic variants and explain part of the individual-specific protein levels in addition to lifestyle and life history (26). However, many proteins have individual-specific levels that are modifiable by lifestyle changes, disease, medication, and even preanalytical processing, adding uncertainty regarding identifiability (27, 28, 29).

### Individuals Can Be Identified by Allelic Information

MS-based proteomics identifies peptides by matching experimental to theoretical spectra calculated from protein sequence databases. Therefore, the proteomics community relies heavily on protein sequence data and associated metadata supplied by consortia such as UniProt (30). As a default to reduce redundancy, UniProt provides all the proteins encoded by one gene as a “canonical sequence” that is usually the most prevalent or likely form. These can be downloaded as FASTA files (human, May 2019). In the case of well-studied species, UniProt provides additional information about variant sequences, including single amino acid polymorphisms from nonsynonymous single nucleotide polymorphisms (SNPs) or polymorphisms from multinucleotide exchange. We generated a human variant FASTA file from UniProt enabling us to identify polymorphisms using the Swissknife PERL module (Experimental Procedures).

In principle, the generated data could uniquely identify individuals using the combination of variant peptides in different proteins. They could also be used to link such variants to disease risks by connecting proteins to the UniProt–Swiss-Prot protein knowledgebase. It currently contains a “human polymorphisms and disease mutations index,” describing 30,706 disease variants, 40,091 polymorphisms without disease implications, and 8085 variants of uncertain medical significance.

To assess a database search with the variant FASTA (a sequence file derived from the database), we revisited the weight loss study data set again. The complete study resulted in 5888 and 6094 peptides when searching against the canonical and the variant FASTA file, respectively, containing 134 and 340 unique peptides. The unique sequences for the canonical FASTA might be explained by the larger search space of the variant FASTA and a general variation due to the MaxQuant search algorithm.

Similar to the unblinding experiment described above, we aimed to extract a panel of peptides from the variant FASTA file experiment, which would allow us to gain additional confidence in the identification of individuals. Peptides were only considered if at least two alleles represented by one peptide for each allele were identified for a protein, resulting in 83 peptides. Additionally, we combined information concerning allele-specific peptides with missed cleavages with the fully cleaved peptide. We further applied a filter step to exclude peptides with very strong variation in their identification (Experimental Procedures).

**Fig. 2:**
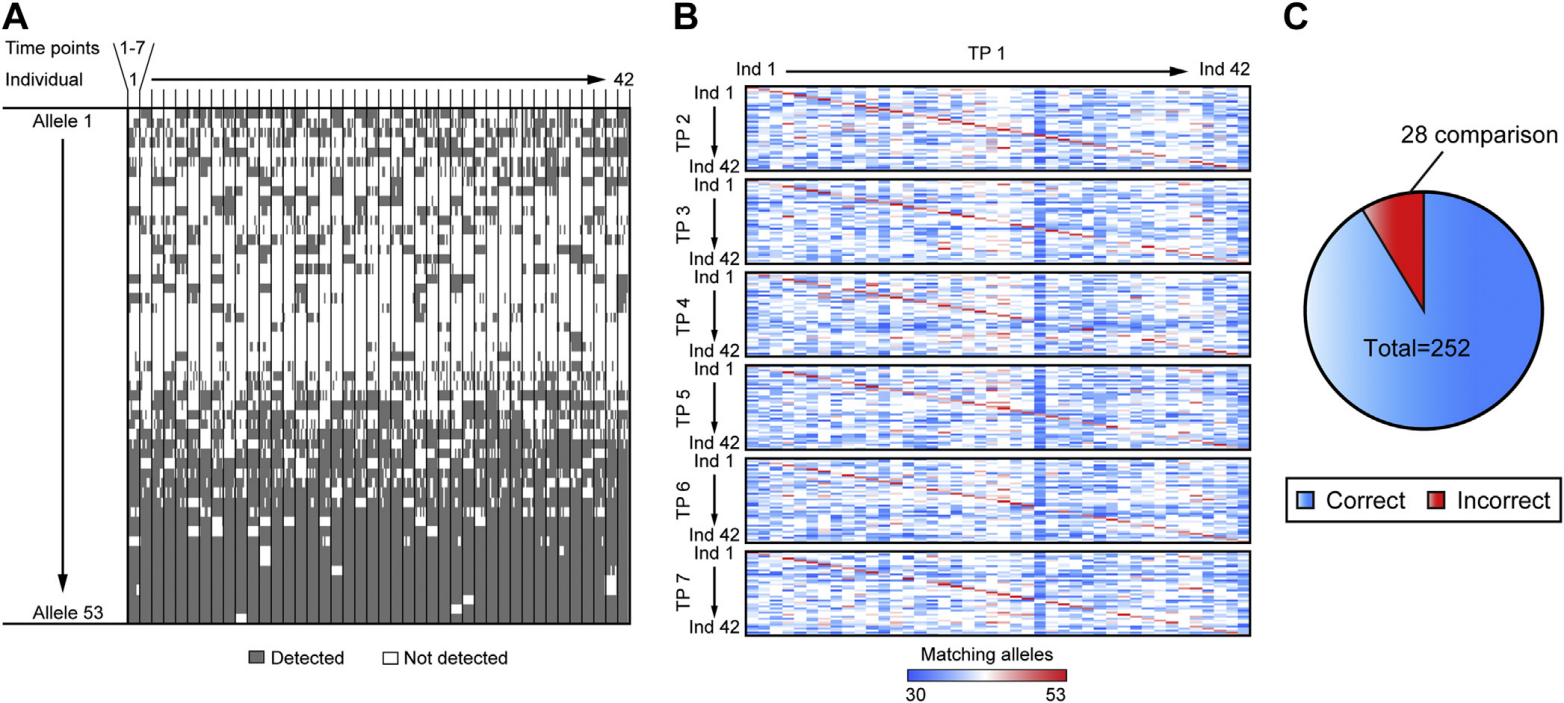
**Allele information in the plasma proteome.***A*, the *grayscale* indicates which peptides were detected across the seven time points (TP) for the 42 individuals in the weight loss study of the 53 variants considered. *B*, heat map for the number of matching present or absent variant peptides between individuals at the first time point (TP 1) and all individuals on the other time points. The *red diagonal lines* reflect the high number of matches of the same individual in adjacent time points. *C*, proportion of correctly identified individuals in the 252 comparisons.

This resulted in a set of 53 peptides of which the allele pattern can be seen across the 42 individuals and the seven time points of the weight loss study (Fig. 2*A*; supplemental Table S1). Next, we identified the study participants by a simple calculation of matching present and absent variant peptides, utilizing the allele information contained in their proteome. Figure 2*B* shows the comparison of each sample to all time point (TP) 1 samples. The median number of matches between two samples within the same individual was 46 and 37 between different individuals. One individual strongly separated from the rest of the population with a median of only 33 matches to the other individuals (blue vertical line in the heat map, Fig. 2*B*). The correct samples were identified in 89% of all comparisons (Fig. 2*C*). In 4998 of 5166 pairwise comparisons matching was correct (1.5% error rate).

The semistochastic sampling of peptides—especially in data-dependent acquisition (dda)—results in missing values of peptides, which decrease the probability of correctly reidentifying an individual. It follows that both data-independent acquisition (dia) and greater data completeness (for instance, in tissue measurements) would result in higher certainty. Note that our calculations are only a proof-of-principle at this point and more advanced experiments including larger and more representative populations have to be done. These issues are and the influence of laboratory errors, which are a frequent issue in DNA technology in forensic science, will have to be taken into account to calculate the true likelihood of reidentifying an individual by variant peptides (31, 32).

### Untargeted Plasma Proteomics Delivers Incidental Diagnostic Findings

As clinical proteomics generates a broad overview of protein levels in a sample, it typically reports on many more conditions than the one under investigation, making “incidental findings” an inherent feature of the technology. If they are related to diseases, this may be sensitive information that raises ethical issues. When dealing with incidental findings, a clear line needs to be drawn between the medical benefits that can be obtained through their return and the principle of respecting an individual’s capacity to choose for themselves whether they wish to have information returned or not. The latter is obtained by informed consent prior to participation in a study or a medical test.

**Fig. 3:**
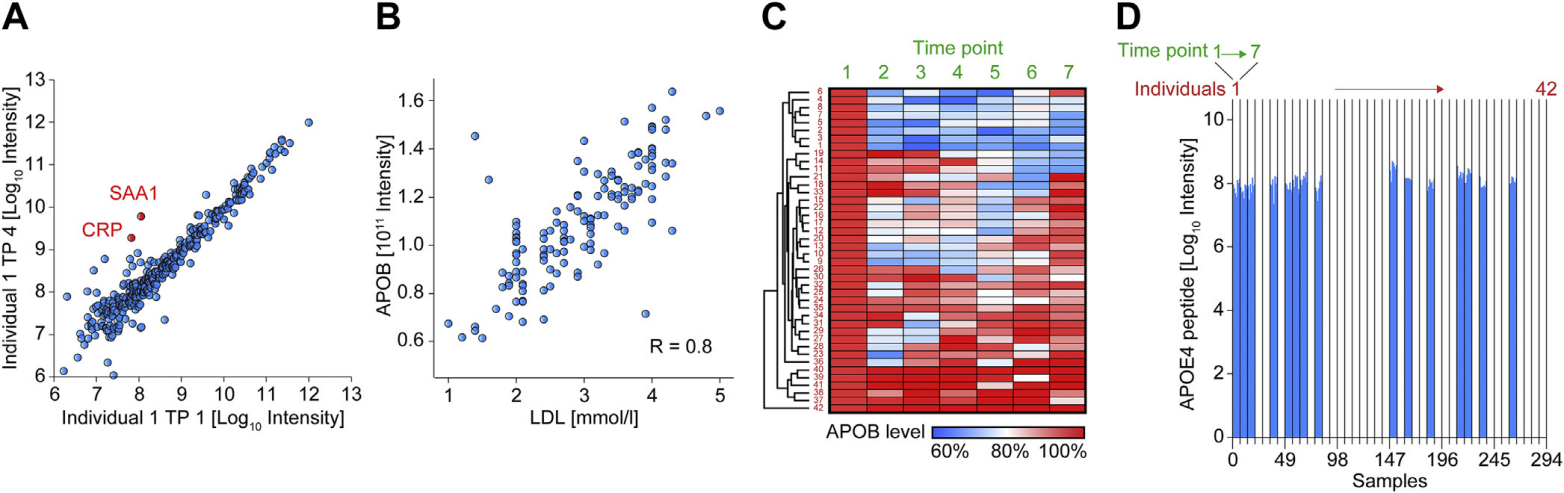
**Disease diagnostics and disease risk assessment relevant proteins.***A*, comparison of the plasma proteomes of one individual, indicating an infection at time point 4 (TP 4). *B*, correlation of LDL levels and proteomic measurements of APOB. *C*, hierarchical clustering illustrating individual-specific responses of APOB levels to weight loss and weight maintenance over seven time points (1, 2, 3, 4, 5, 6, 7). *D*), intensities of the quantified APOE4 allele determining peptide across individuals.

Even short 20 min MS-based proteomics measurements quantify about 50 proteins that were approved by the U.S. Food and Drug Administration (FDA) as biomarkers, resulting in a multilayer reflection of the human health state (14). These MS-quantified markers include C-reactive protein (CRP), which reports primarily on inflammation and is one of the most frequently requested protein measurements in clinical practice. The coregulated protein serum amyloid alpha 1 (SAA1) shows highly similar fold changes upon infection and is likewise covered (Fig. 3*A*), resulting in a more inclusive reflection of an individual’s inflammation status than the routine assessment of CRP alone. While an infection is usually short-term and benign, other MS-identifiable proteins may indicate life-threatening diseases, such as the cancer marker MUC16.

The health consequences of diabetes, one of the leading causes of death worldwide, can be minimized if the disease is diagnosed early and managed properly (33). In clinical practice, diagnosis of diabetes involves measuring HbA1c, the glycated form of hemoglobin. The concentration-dependent Maillard reaction of sugars with the amine groups of proteins is responsible for this glycation. In addition to hemoglobin, all other blood proteins and in particular the long-term circulating high-abundant ones are also glycated. In our experience, hundreds of glycated peptides are easily detectable in all plasma proteome profiling experiments (14, 16, 34). Their levels are an indicator of prediabetic or diabetic status, which is particularly relevant as up to a third of the population that has these conditions are not aware of it.

Apart from acute disease, proteomics covers proteins connected to the risk of a future disease. The lipid homeostasis system is covered by more than 20 factors (16), several of which—including apolipoprotein A1 (APOA1), apolipoprotein B (APOB), and apolipoprotein(a) (LPA)—are important predictors of cardiovascular diseases (35). MS-derived APOB intensities correlate to LDL-levels as shown in a data set of 142 samples in our weight loss study (Fig. 3*B*). This indicates that these apolipoproteins can be used to determine risk of cardiovascular diseases similarly to cholesterol, for which they are carriers. It is well known that decreasing high cholesterol levels by lifestyle changes or medication is beneficial for health outcomes (15, 35). Thus, an apolipoprotein panel derived from plasma proteomics likely provides at least the level of actionable health information than a routine cholesterol test. Furthermore, as individuals may respond quite differently to various treatments as shown in the regulation of APOB levels upon weight reduction (Fig. 3*C*), proteomics may provide more individualized and detailed information. This is an example where the risk is known and treatment options to significantly reduce future medical conditions may be significantly improved by proteomics.

MS-based proteomics can also report on risks for which no treatment option is currently available, making them “non-actionable.” For instance, the three APOE alleles—APOE2, APOE3, and APOE4, can be differentiated by sequence specific peptides. Knowledge concerning the status of the APOE4 allele (7.5–15.6% of the population), which strongly increases risk of Alzheimer’s disease, is medically unactionable information as there is currently no available treatment (36). Return of such severe unactionable information could leave some individuals with psychological trauma, frustrate others, and might potentially negatively influence future personal decisions in a negative manner. The APOE2 allele (6.7–10.0% of the population) increases cholesterol levels and cardiovascular pathologies (36). In contrast to APOE4, this knowledge is actionable and could lead to the decision to take cholesterol lowering medication or dietary interventions to decrease cardiovascular disease risk. There are therefore much more persuasive reasons for the return of information concerning APOE2 status.

### Untargeted Plasma Proteomics Delivers Personally Sensitive Findings

**Fig. 4:**
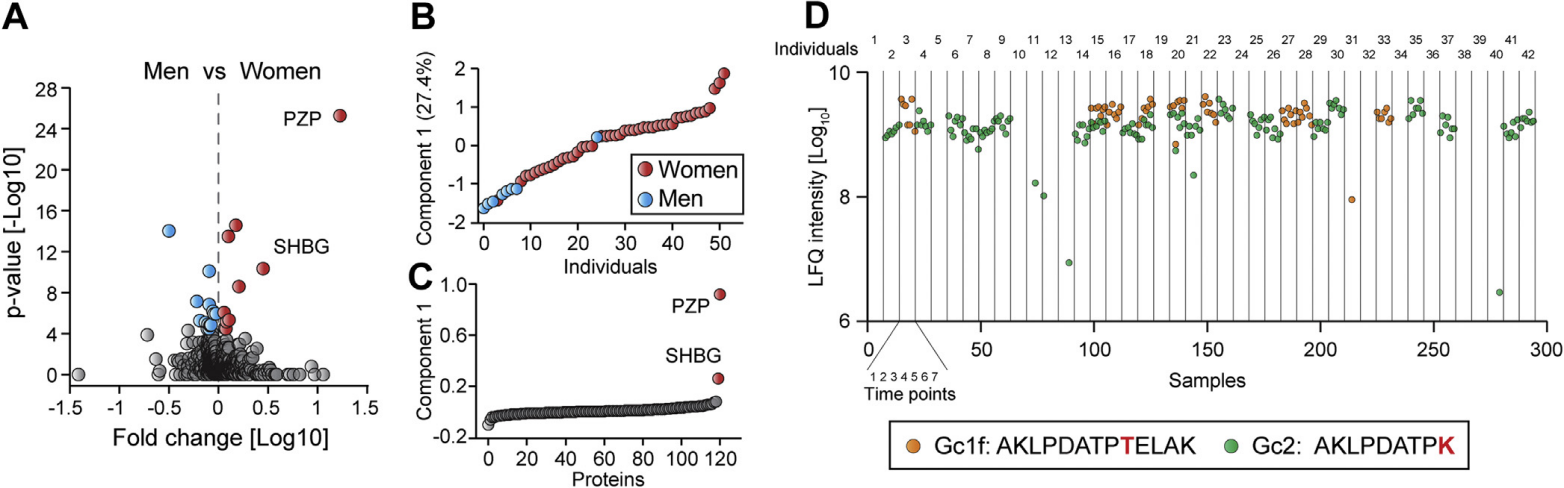
**Information with the potential to discriminate individuals.***A*, comparing the plasma proteomes of all individuals and samples in the weight loss study (15). Proteins with elevated levels in women and men are highlighted in *red* and *blue*, respectively. *B*, one-dimensional principal component analysis for plasma samples at one time point. *C*, proteins and their distribution to the separation in (*B*). *D*, vitamin D-binding protein determining alleles quantified by MS-based proteomics.

The recognition of the equal worth and dignity of all members of the human family as enshrined in universally adopted and near-universally accepted normative and legal codes globally represents the outcome of centuries of struggle for legal and moral equality before the law and society. These efforts aim at stopping and preventing discrimination, which refers to unequal treatment on the basis of various morally irrelevant attributes such as gender, race, color, or national or ethnic origin, all of which still occur in both explicit and implicit forms in modern society. Therefore, it is morally noteworthy that one can distinguish the proteomes of men and women and of reproductive status (14). Comparing the longitudinal proteomes of women and men of our weight loss study revealed that the levels of estrogen-regulated proteins such as the sex hormone binding globulin (SHBG) and the pregnancy zone protein (PZP) were significantly elevated in women (Fig. 4*A*). While the levels of these proteins depend on several additional factors such as age and lifestyle, they are clear indicators of gender. Applying a one-dimensional principal component analysis clustered almost all women and men separately, although one of the eight men and one of the 34 women were assigned to the opposite clusters due to untypical PZP levels (Fig. 4, *B* and *C*). It is known that SHBG and especially PZP increase more than tenfold during pregnancy. Additionally, there are highly specific measures for determining pregnancy by quantifying placental proteins such as the family of pregnancy-specific glycoproteins (PSGs), which are the most abundant trophoblastic proteins in maternal blood during pregnancy (37, 38), and they are in our experience readily detectable by MS-based proteomics. Their concentration can increase over 1000-fold, even exceeding placental peptide hormone human chorionic gonadotropin (hCG), which is usually determined in pregnancy tests. This is significant given that pregnancy status is information that individuals often seek to keep private, as much of the discrimination faced by women is related to the unique status of (potential) motherhood.

Given that body weight is connected to discrimination, we investigated the weight loss study for pertinent markers. Interestingly, SHBG and proteins of the innate immune system that we readily quantified are among the proteins most affected by body mass (15, 16). Although individual-specific differences, acute inflammation, and gender might influence these parameters, they could conceivably be used to predict a person’s weight based on their plasma proteome or more pertinently likely negative health effects for that person.

Similarly, it is also possible to discern information regarding the ethnic background of an individual using alleles in a similar way as described above (39). Furthermore, ethnic differences can complicate analytical results. A prominent example of this is the abundant plasma protein vitamin D-binding protein (GC), which has three common alleles Gc1f, Gc1s, and Gc2 with very different allele distribution depending on ethnic background. Gc1f is most frequent in West Africans and African Americans and least common in Caucasians (40, 41). All three alleles should result in potential MS-detectable peptides, and we identified the Gc1f peptide in nine and the Gc2 peptide in 21 of 42 study participants at each of the seven time points (Fig. 4*D*). However, the third peptide was not detectable, presumably due to poor ionization.

### How to Render the Plasma Proteome Ethically Unproblematic and General Data Protection Regulation (GDPR) Compliant

The legal act of the European Union for the protection of personal information—the General Data Protection Regulation (GDPR)—regulates data protection and privacy with one of the central aims of giving control of personal data to the corresponding individuals (Directive 95/46/EC, (42)). Implemented in 2018 across the EU, GDPR supplies rules relating to the protection of an individual with regard to the processing of personal data and to the movement of personal data. It also regulates flow of information into “third countries,” outside the sphere of GDPR. In the United States, the HIPAA (Health Insurance Portability and Accountability Act) Privacy Rule is a similar directive on data protection. However, HIPAA focuses on the protection of individually identifiable health information, whereas GDPR is a regulation on all data that can be related to a person. Furthermore, there is an international trend toward GDPR style rules across many jurisdictions.

Study participants and patients must be informed about the potential information content of their proteome in advance of study participation. Ideally, they would be given the opportunity to state whether and to what extent their data can be used for the benefit of research and third parties. Furthermore, individuals should be informed about the potential and range of incidental findings and their preferences as to return of such information should be ascertained. However, in studies this is only possible under specific circumstances, for example, where data is pseudonymized or nonanonymized instead of anonymized. However, nonanonymization of samples and data collides with the protection of personal data as the proteome contains sensitive information about individuals and therefore adequate rules have to be promulgated and adhered to when working with this kind of data in research and clinical contexts. In genomics, samples and data from individuals have to be pseudonymized before analysis to comply with the GDPR. Pseudonymization still allows for the linking of an individual to their data under specific conditions and would act as a safeguard against third-personal access to personal and sensitive information. In contrast, this would not be possible in anonymized data and would hinder to link new studies and their results to previous findings.

Clearly, individuals have to decide to what extent their samples and personal data should be processed, and they have to be aware of the range of information that can be extracted. Informed consent must be given, which can happen either in an opt-out or opt-in modality. The choice between these will have a significant impact on the extent of information available for research and processing (see our accompanying paper, (21)). These issues exist along spectra of severity, such that their associated ethical questions do not admit of binary answers and degrees of likelihood, risks, benefits, and harms must be considered. Next to organizational measures and access to the samples and data, the extent further analysis, storage, and integration of proteomics data for research and clinical purposes are a topic of great importance. In proteomics, mass spectra of peptides are generated and saved in raw data files. In principle, allele-specific peptides could be deleted from the raw data, but this would itself go against good laboratory practice, which strongly discourages data manipulation. However, the sharing of raw data is beneficial due to several reasons including general research reproducibility. Therefore, in the research context instead of having controlled access to raw data, data-sanitization procedures might be applied to have the optimal trade-off between data utilization and privacy protection as it has recently been proposed in genomics (43). To prevent the existence of such data, one could instead direct data acquisition accordingly, which would be difficult or impossible for data-independent acquisition (dia), challenging for data-dependent acquisition (dda) and easily accomplished for targeted proteomics.

Once data is acquired, the interpretable information still depends on the processing workflow. For example, allele information is easily extracted when a sequence database (FASTA) file with allele information is used. FASTA files without allelic information would render the proteomic readout nonidentifiable for many purposes, as protein levels alone cannot identify an individual to the degree of certitude necessary in forensic and similar contexts. Therefore, tightly restricting access to the raw data might be warranted in many circumstances. We suggest that in clinical environments, the handling and analysis of data should be done in an automatic fashion. Access could be secured by technologies such as blockchain, ensuring that only approved information is read out and reported to the patient, depending on their former written consent.

MS-based proteomics is uniquely information-rich because it accurately and quantitatively measures thousands of peptides in clinical samples. However, affinity-based methods are also increasingly applied to the plasma proteome (26). As only a single or few epitopes are probed, there is much less chance of revealing allele-specific information. Furthermore, preanalytical factor such as freeze–thaw cycles can affect epitope recognition, whereas this does not introduce variations in MS-based methods (29). Thus, identifiability is currently not a particular issue in affinity-based methods, and incidental findings could be stripped out by removing them from the reported protein level measurements.

## Discussion

Exploring the plasma proteome in various clinically relevant areas, we have encountered several categories of findings with potential ethical implications. In this paper, we systematically investigated the main ones using a plasma proteome profiling study (15), which served as a prototypical case. Quantitative protein levels allowed us to detect acute diseases and markers for future events that raise ethical issues. Individual-specific levels of plasma proteins enabled us to identify individuals in this longitudinal study. We further showed that MS-based proteomics delivered broad knowledge about allele distribution with the potential to identify individuals, predict disease risks, and detect alleles characterizing ethnic groups. Even though insights based on allele variation are not very extensive in plasma yet, they will become more so through further technological progress. This is already the case for the proteomic analysis of tissue samples because tens of thousands of peptides are routinely measured and large-scale efforts are already on the horizon (44, 45, 46, 47). Much remains to be learned about plasma proteome alterations in response to variables such as lifestyle, disease, and medication. If these variables change the plasma proteome, they might complicate the identification of an individual. However, the continuous exploration of the plasma proteome will increasingly allow us to acquire information about plasma proteome modulators and take them into account. Moreover, as technology progresses, it will enable the investigation of increasingly large studies while expanding the information that can be extracted from a single plasma proteome.

As the power of proteomics increases (11), so do insights that may be derived from an individual’s sample. Indeed this is necessary for the medical application of proteomics. Yet the hypothesis-free nature of proteome profiling also yields information not directly relevant to the study or intervention at hand, which may nevertheless be relevant for other medical reasons or for other purposes entirely. Since not all uses of this additional information are benign, it is important to discuss how the benefits of additional knowledge can be reaped, maximized, and shared, while avoiding the potential for harm and exploitation which that knowledge may also bring.

These almost exclusively relate to the knowledge that can be obtained from proteomic data and thus have much in common with issues familiar from broader health and research contexts such as data storage, data sharing, and adequate consent. However, the proteomic context is unique, and the progress made in other fields does not necessarily translate easily. Therefore, it is important that the proteomics community is aware of, raises, and discusses these issues to protect data subjects and facilitate research in ethical and legal ways.

We hereby wish to start a discussion within our proteomics community about potential uses of the data in our hands. We believe this is important not only to maximize benefit and minimize harm, but crucially also because our community is best positioned to explore questions not only of an ethical and legal nature but are also intertwined with technical and scientific issues related to the reliability, accuracy, storage, sharing, propriety, and kinds of inferences that may be surmised from proteomics.

We hope this discussion can serve as a first step toward serious scientific self-regulation in the proteomic community, which is acceptable to regulators and society, as has happened with recombinant DNA techniques at the Asilomar conference already in 1975 (48), and as is currently being attempted in the context of editing the human germline (49).

To achieve this, the proteomics community will need to become aware of ethical issues involved and begin to discuss them seriously. Luckily, the effort will not have to be made from scratch as structurally similar issues have been extensively debated in other fields. Nevertheless, these debates are far from settled and the unique research context of proteomics means that potential answers cannot be imported wholesale but rather need to have their fit and applicability assessed and thoroughly discussed.

In conclusion, the increasing power of MS-based proteomics for generating both medical and nonmedical insights brings with it a concomitant increase in the importance of associated ethical issues. We have presented some of these issues by showcasing a single plasma proteomics study. The purpose of this exploratory contribution has been to point out that these issues already exist and to initiate discussion; but more importantly, we hope to stimulate others to point the attentions of ourselves and the community to issues that are not discussed here. In our accompanying paper, we further elucidate and develop ethical implications from a bioethical and philosophical perspective in the hope of starting a discussion concerning the first potential guidelines for the community: (21).

## Data and Material Availability

The MaxQuant output files of the searches have been deposited at the ProteomeXchange Consortium *via* the PRIDE partner repository and are available *via* the identifier PXD021677.

## Conflict of interest

The authors declare that they have no conflicts of interest with the contents of this article
