# Supplementary figures and images for: Plasma Proteomes Can Be Reidentifiable and Potentially Contain Personally Sensitive and Incidental Findings
Source: Mol Cell Proteomics. 2021 Jan 11;20:100035. doi: 10.1074/mcp.RA120.002359 (PMC7950134; doi:10.1074/mcp.RA120.002359)

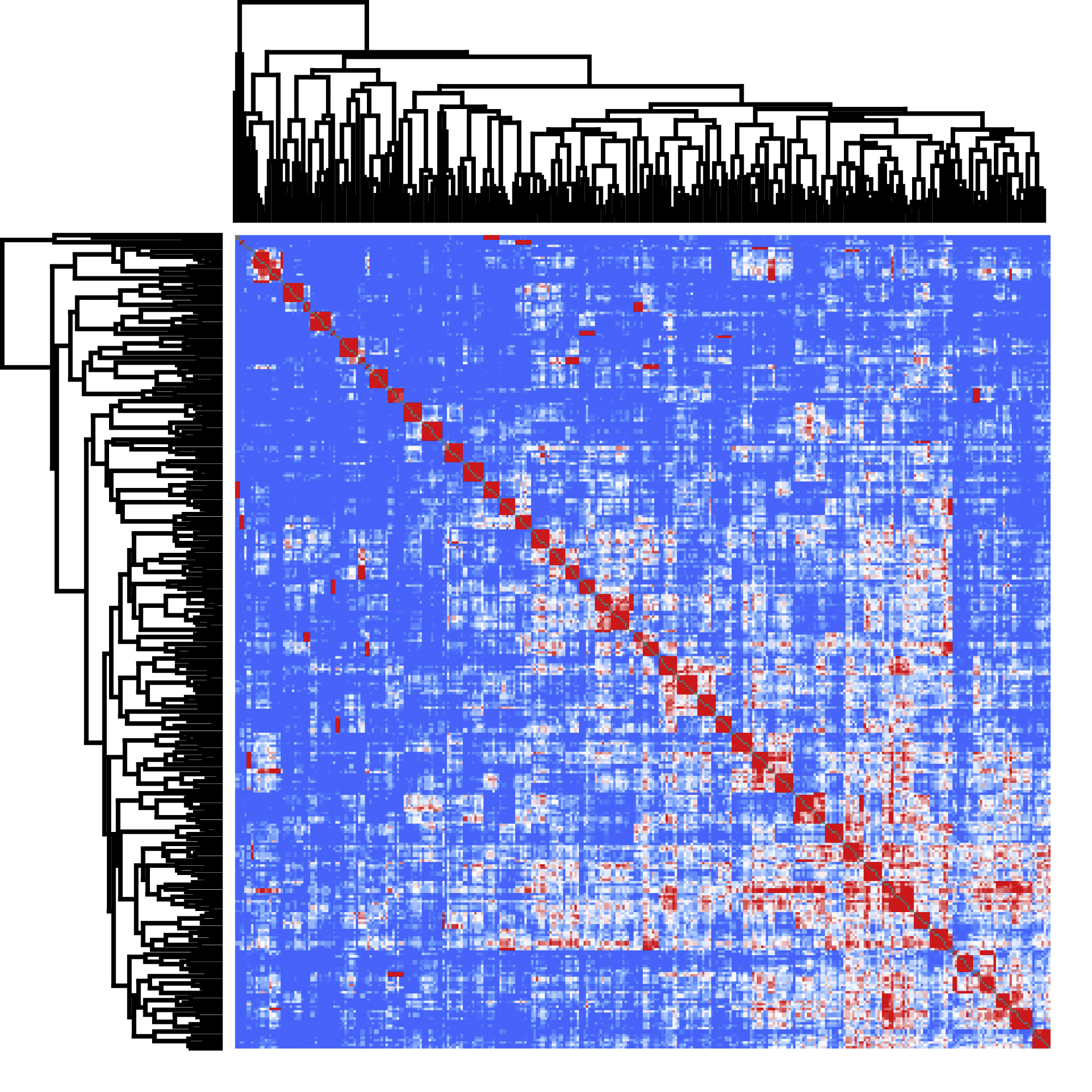

Supplement: Supplemental Fig. S1 [file figs1.jpg]
